# Controlling Harmful Cyanobacteria: Taxa-Specific Responses of Cyanobacteria to Grazing by Large-Bodied *Daphnia* in a Biomanipulation Scenario

**DOI:** 10.1371/journal.pone.0153032

**Published:** 2016-04-04

**Authors:** Pablo Urrutia-Cordero, Mattias K. Ekvall, Lars-Anders Hansson

**Affiliations:** 1 Department of Biology, Lund University, Ecology building, SE-223 62 Lund, Sweden; 2 Center for Environmental and Climate Research, Lund University, Ecology Building, SE-223 62, Lund, Sweden; University of Connecticut, UNITED STATES

## Abstract

Lake restoration practices based on reducing fish predation and promoting the dominance of large-bodied *Daphni*a grazers (i.e., biomanipulation) have been the focus of much debate due to inconsistent success in suppressing harmful cyanobacterial blooms. While most studies have explored effects of large-bodied *Daphnia* on cyanobacterial growth at the community level and/or on few dominant species, predictions of such restoration practices demand further understanding on taxa-specific responses in diverse cyanobacterial communities. In order to address these questions, we conducted three grazing experiments during summer in a eutrophic lake where the natural phytoplankton community was exposed to an increasing gradient in biomass of the large-bodied *Daphnia magna*. This allowed evaluating taxa-specific responses of cyanobacteria to *Daphnia* grazing throughout the growing season in a desired biomanipulation scenario with limited fish predation. Total cyanobacterial and phytoplankton biomasses responded negatively to *Daphnia* grazing both in early and late summer, regardless of different cyanobacterial densities. Large-bodied *Daphnia* were capable of suppressing the abundance of *Aphanizomenon*, *Dolichospermum*, *Microcystis* and *Planktothrix* bloom-forming cyanobacteria. However, the growth of the filamentous *Dolichospermum crassum* was positively affected by grazing during a period when this cyanobacterium dominated the community. The eutrophic lake was subjected to biomanipulation since 2005 and nineteen years of lake monitoring data (1996–2014) revealed that reducing fish predation increased the mean abundance (50%) and body-size (20%) of *Daphnia*, as well as suppressed the total amount of nutrients and the growth of the dominant cyanobacterial taxa, *Microcystis* and *Planktothri*x. Altogether our results suggest that lake restoration practices solely based on grazer control by large-bodied *Daphnia* can be effective, but may not be sufficient to control the overgrowth of all cyanobacterial diversity. Although controlling harmful cyanobacterial blooms should preferably include other measures, such as nutrient reductions, our experimental assessment of taxa-specific cyanobacterial responses to large-bodied *Daphnia* and long-term monitoring data highlights the potential of such biomanipulations to enhance the ecological and societal value of eutrophic water bodies.

## Introduction

The excessive growth of undesirable plant and algal populations is a ubiquitous phenomenon in nutrient-rich marine and freshwater ecosystems [1–2]. A classical example of this is the widespread incidence of harmful cyanobacterial blooms as a result of the eutrophication and likely warming of water bodies [3]. Massive proliferations of cyanobacteria have strong impacts on food web interactions and ecosystem function, as well as on ecosystem services, through increased hypoxia and ‘dead zones’ [4]. In addition, numerous cyanobacterial taxa produce toxins with considerable risk to human and animal welfare [5] and impose substantial economic costs to human societies [6]. Despite ongoing attempts to control harmful cyanobacterial blooms, there is still an urgent need to find sustainable methods to control their frequency and magnitude [3]. This study explores taxa-specific responses of cyanobacteria to the grazer control by large-bodied *Daphnia* and ultimately provides guidance towards better restoration practices in eutrophic waters.

The use of herbivorous zooplankton to control cyanobacterial growth has been the focus of much debate in the ecological literature [7,8]. On the basis of food web theory, zooplankton abundance and community structure can be altered through the removal of planktivorous fish, thereby reducing predation on zooplankton and increasing top-down pressure on phytoplankton [7,9]. In contrast, cyanobacterial blooms often persist during these ‘biomanipulations’ and the underlying causes are still not fully understood [8,10,11]. Several studies have shown that the large size and specific morphology of many species of cyanobacteria (single cells growing as filaments and colonies) may provide a size refuge to zooplankton grazing [12]. In addition, many cyanobacterial taxa produce toxic metabolites and have low nutritional value, thereby reducing growth and fitness of herbivore communities [13–15]. Hence, it has been argued that the evolution of these cyanobacterial defenses ultimately determines the capacity of herbivores to regulate their population dynamics [8,16].

Maximum zooplankton herbivory is generally not achieved in natural environments due to fish predation [17], which makes it difficult to quantify the potential of large, efficient zooplankters to regulate the growth of cyanobacteria. For example, body-size is a critical trait shaping consumer-prey interactions and size-dependent predation by fish preferentially eliminates large-bodied grazers, such as the crustacean *Daphnia* [18]. Large-bodied generalist grazers like *Daphnia* have higher grazing rates than smaller-bodied zooplankters (e.g., copepods and small cladocerans) and may dominate plankton communities at low levels of fish predation [17]. While studies have explored the top-down effects of large-bodied *Daphnia* as a mean to control cyanobacterial growth in eutrophic water bodies (e.g., [19–22]), most have generally focused on cyanobacterial responses at the community level and/or on only few dominant species, thereby providing little information on the vulnerability to grazing of specific taxa in diverse cyanobacterial communities. As a result, we still need to further our understanding of whether maximizing *Daphnia* herbivory by reducing fish predation can overcome the wide range of cyanobacterial defenses to grazing, and whether other means of restoration proven powerful to reduce cyanobacterial growth, such as nutrient reduction, should also be applied in parallel [7,8]. In addition, only a few studies have assessed seasonal zooplankton-cyanobacteria interactions that integrate a significant part of the cyanobacterial diversity and species succession naturally occurring in aquatic ecosystems [8].

To address these questions, we used a set of *in situ* experiments in a eutrophic lake with occurrence of highly diverse cyanobacterial blooms to investigate seasonal taxa-specific responses of cyanobacteria to large-bodied *Daphnia*. In addition, we monitored the lake (Lake Ringsjön), a large, eutrophic lake that was subjected to long-term fish removal (biomanipulation), as a proof of concept for the increased efficiency of zooplankton herbivory through higher *Daphnia* abundances and larger body-size. Our study follows experimental results and field data presented in previous studies by [23,24]. These studies provided important information on 1) the responses of cyanobacteria at the community level and cyanotoxin levels to grazing by large-bodied *Daphnia*, 2) the contribution of the natural zooplankton community (copepods and small cladocerans) to grazing of specific cyanobacterial species, and 3) the temporal variation of cascading effects from the removal of fish on total cyanobacterial biomass and toxin levels in the lake. Here we extend those results by investigating cyanobacterial responses to grazing by large-bodied *Daphnia* below the community level, thereby mimicking the expected vulnerability of cyanobacteria at different taxonomic levels in a desired biomanipulation scenario, where fish removal and recovery of large-bodied *Daphnia* are successful [7,17]. In addition, our lake monitoring data evaluates the effects of biomanipulation on native *Daphnia* populations by using a recently available longer data set than previous studies in this lake [23,24], as well as investigates the potential cascading effects on specific bloom-forming cyanobacterial taxa. Therefore, the combination of both experimental and field data aims to evaluate the efficiency of restoration practices based on the control of cyanobacterial growth by *Daphnia* herbivory. Based on the efficiency and generalist-feeding mode of large-bodied *Daphnia* [8], we hypothesized a strong suppression of the growth of most cyanobacterial taxa in our experiments, regardless of cyanobacterial densities, morphologies features and putative toxicity. In addition, we expected the biomanipulation in the lake to boost substantially the abundance and body-size of native *Daphnia* and to reduce the biomass of the most dominant cyanobacterial taxa.

## Material and Methods

### Grazing experiments

Our study was conducted in the eutrophic Lake Ringsjön (55° 52´ 28´´ N, 13° 39´ 53” E), southern Sweden, which consists of three interconnected basins with a total surface area of 40 km^2^. The climate is southern Sweden is humid all year around, with cool and windy winters and mild summers. Nutrient levels increased in the 1960s and 1970s because of the intensification of agricultural practices and urbanisation [25]. Since then there have been regular blooms of potentially toxic cyanobacterial taxa such as *Aphanizomenon*, *Dolichospermum*, *Microcystis* and *Planktothrix* [25]. Three grazing experiments were performed in the western basin of Lake Ringsjön (55° 52´ 57´´ N, 13° 27´ 5” E), in June, July and August 2012. These experiments investigate the responses of cyanobacteria at different levels of taxonomic resolution and extend the results by [23], who focused on the effects of *Daphnia* on cyanobacteria at the community level. The experiments followed the standard methods described by [26], which have been applied by many others in both field and laboratory experiments [27–29]. The experiments were conducted in transparent plastic containers with a maximum volume capacity of 10 L. The six 10 L containers were filled with 9 L of filtered (150 μm mesh) lake water, containing the natural phytoplankton community in the lake, but excluding grazers larger than 150 μm in body-size. The zooplankton used in the experiments, *Daphnia magna* (mean size ± SD: 1752 ± 377 μm, mean individual biomass ± SD: 26 ± 13 μg; measured from the eye to base of the tail) originated from a population (with unknown record of their genetic diversity) isolated from the eutrophic shallow lake Bysjön (55° 40´ 31´´ N, 13° 32´ 43” E) (see [14] for more information about this lake). These *Daphnia* were reared in the laboratory at 25°C and fed with a mixture of phytoplankton (green algae, cryptophytes and cyanobacteria) over several months. We added the *Daphnia magna* to the six 10 L containers in an abundance gradient of 0.25, 0.5, 1, 2, 4 and 6 times 8±4 *Daphnia magna* per liter (which lies within the range of natural abundance of native *Daphnia* (approximate individual biomass: 5 ± 3 μg) typically found in Lake Ringsjön), thereby successfully generating a gradient of biomass of *Daphnia magna*. The biomass gradients included other zooplankters (e.g., copepods and small cladoceran) from the *Daphnia* culture, but their relative biomass proportion was negligible (<2%) compared to that of *Daphnia*. Although the 150-μm mesh cannot retain grazers smaller than 150 μm, their initial abundances remained the same across all the enclosures and any changes in the phytoplankton community structure could therefore be exclusively attributed to changes in the introduced *Daphnia*. The containers were sealed (so that no exchanges were made possible between the containers and surrounding environment), closed to the atmosphere and placed 1 m apart in the surface water of the lake (i.e., in the photic zone). Hence, the containers were equally exposed to the same natural temperature and light climate regime as in the lake.

Samples for cyanobacterial counts and chlorophyll-*a* analyses (used as a proxy of total phytoplankton biomass) were taken before and after 3 days of incubation in the lake according to [26]. Samples for *Daphnia* counts were taken at the end of the experiment by filtering the entire volume (9 L) of the containers through a 150-μm mesh. Chlorophyll-a samples (50 mL) were immediately filtered through GF/C filters (Whatman, 25 mm) and the filters were frozen until further analysis. The chlorophyll-*a* concentration was determined with 3 mL ethanol (96%) extractions and measurements on a spectrophotometer (Shimadzu, UV-2600) [30]. Samples for cyanobacterial and *Daphnia* counts (100 mL) were immediately fixed in Lugol’s solution after sampling and stored in a cold room at 4°C. Cyanobacteria were counted at different levels of taxonomic resolution (species, genus and class) on a Olympus CK40 (LRI instrument AB, Lund, Sweden) inverted microscope and biomasses were estimated according to [31]. The size of each cyanobacterial species was estimated by measuring the length of filaments and the spherical diameter of colonies. The abundance of *Daphnia* were determined using a stereoscopic microscope (Olympus SZ40) at x20 magnification, and biomasses were estimated using length-weight regressions according to [32] and [33].

Responses of total phytoplankton biomass and cyanobacteria at different levels of taxonomic resolution (species, genus and class) to grazing were determined by linear regression analyses (F-test, n = 6) with algal net growth rate (*r*) as the dependent variable and the *Daphnia* biomass as the independent variable according to [26]. Algal net growth rates (*r)* were calculated as *r =* ln(N_t_/N_0_)/Δt, Δtwhere N_0_ and N_t_ express the algal biomass (mg L^-1^ for microscopic counts or μg L^-1^ for chlorophyll-*a* values) at the beginning and after the end of the 3 days incubations (represented by Δt). All data was analysed with SPSS 21 for Macintosh and plots were created with GraphPad Prism. No specific permits were needed to conduct these experiments, which did not involve any endangered or protected species.

### Field study

The first biomanipulation attempt in Lake Ringsjön was between 1989–1992 [7], but the lake started to show signs of degradation again from the mid 90s. In 2005 a new biomanipulation was initiated by trawling for planktivorous and benthic fish, mainly targeting roach (*Rutilus rutilus*), bream (*Abramis brama*) and small perch (*Perca fluviatilis*) [23]. Lake chemical variables (total phosphorus and phytoplankton-chlorophyll-a), and zooplankton and cyanobacterial community structure have been monitored since 1996 in the western basin of Lake Ringsjön (mean depth: 3m; maximum depth: 5.4 m). Samples were taken monthly from April to October each year at the location of maximum water depth in the lake. An integrated sample (10–30 L) of the water column was taken with a Plexiglas sampler from which subsamples for chlorophyll-*a*, cyanobacterial counts and total phosphorous were collected. The remaining water was filtered through a 50-μm mesh to collect the zooplankton individuals, which were stored in 100 mL bottles. Cyanobacteria and zooplankton samples were immediately preserved in Lugol’s solution and stored at 4°C after sampling. Chlorophyll-*a*, cyanobacteria and zooplankton analyses were performed by using the same methods described above for the experiments. Total phosphorus samples were frozen until further analyses performed according to [34]. For more information about this biomanipulation programme see the website ‘http://www.ringsjon.se‘(only in Swedish).

We used seasonal means (April-October) of the variables of interest (see below) and divided the data set into years prior to (1996–2004; hereafter named ‘before biomanipulation’) and after the start of the biomanipulation (2006–2014; hereafter named ‘during biomanipulation’). No monitoring data on zooplankton was available for 2002 and 2012, and these years were therefore excluded from the analyses. Pearson´s correlations were used to study changes before and during the biomanipulation in phytoplankton and cyanobacterial community structures in relation to total zooplankton abundances (cyclopoid and calanoid copepods, including nauplii; *Daphnia*; and the small cladocerans, *Bosmina* and *Chydorus*), *Daphnia* abundance, *Daphnia* body-size (μm) and total phosphorous concentrations (μg L^-1^). TN:TP ratios were always above the Redfield ratio (16: 1 mass ratio), except for 2003 (TN: TP ratio = 15.34). This suggests that phosphorous, and not nitrogen, was likely the limiting nutrient throughout the study period. Chlorophyll-a values (μg L^-1^; hereafter denoted as ‘total phytoplankton’) and total cyanobacterial biomass (mg L^-1^) were used as proxies of changes in phytoplankton at the community level. We later analysed the dominant bloom-forming cyanobacterial taxa *Aphanizomenon*, *Dolichospermum*, *Microcystis* and *Planktothrix*. All data was log (x) transformed prior analyses with SPSS 21 for Macintosh and plots were created with GraphPad Prism. No specific permits were needed to conduct this study, which did not involve any endangered or protected species.

## Results

### Grazing experiments

**Fig 1:**
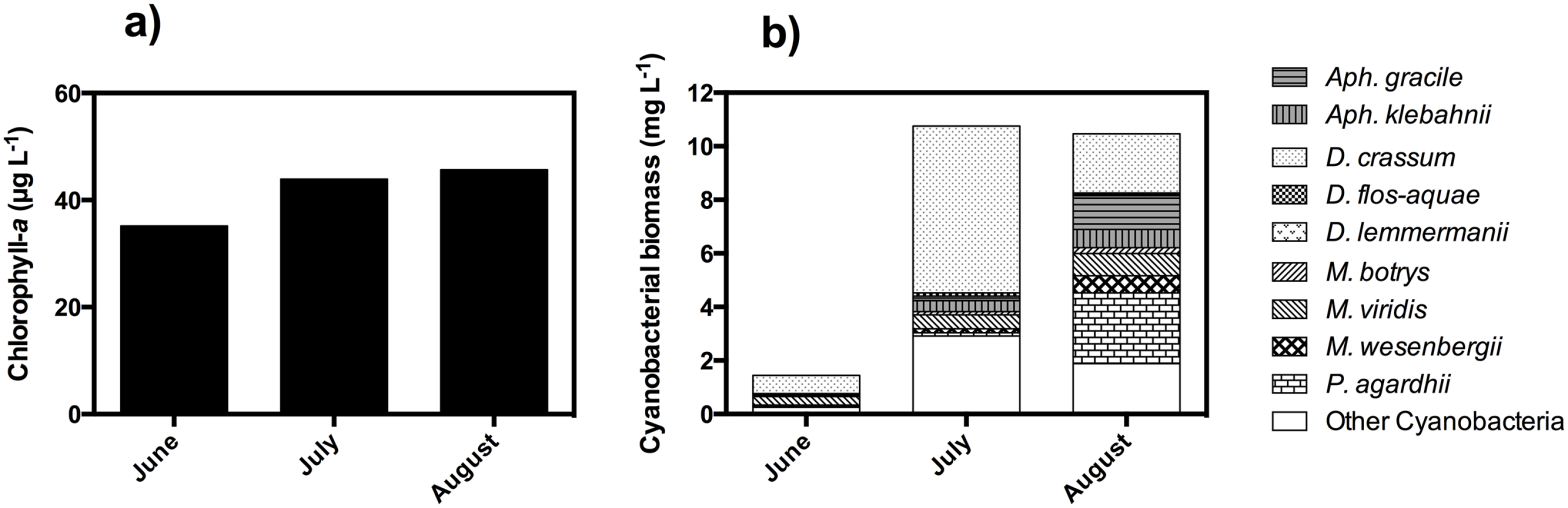
Biomasses of a) total phytoplankton and b) dominant cyanobacterial species used in the grazing experiments (T_0_ = beginning of the experiment). Values are means across the six containers calculated from samples taken previous to the initiation of the experiments.

Chlorophyll-a values were typical for eutrophic conditions throughout the experiments, ranging between 35 (June) and 46 μg L^-1^ (August) on average across all containers (Fig 1). Although we do not present detailed data on other phytoplankton taxa than cyanobacteria, diatoms and green algae dominated the phytoplankton community in early summer. However, cyanobacterial biomass increased considerably from June to July, dominated by the spiral filament-forming *Dolichospermum crassum* (Fig 1). In the August experiment, the cyanobacterial community was diverse and dominated by *Aphanizomenon*, *Dolichospermum*, *Microcystis* and *Planktothrix* species (Fig 1). Only the species *Aphanizomenon gracile* and *Planktothrix agardhii* showed considerable filament-size changes across the three experiments (Table 1). However, there were marked differences in size among *Dolichospermum* species, where the filamentous *D*. *flos aquae* and *D*. *lemmermanii* were substantially smaller than *D*. *crassum* (Table 1). *Planktothrix agardhii* represented the filamentous species with the greatest average length (Table 1). The colony-forming *Microcystis* showed almost no differences in size among the three most abundant species *M*. *botrys*, *M*. *viridis* and *M*. *wesenbergii* (Table 1).

**Table 1:** Morphological characteristics of dominant cyanobacterial species used in the grazing experiments (T_0 =_ beginning of the experiment) in Lake Ringsjön in 2012. Means (± SD) sizes and size ranges of cyanobacterial taxa (n = 30 individuals) are represented by ‘maximum linear dimensions’ (length of filaments and spherical diameter of colonies) in the three grazing experiments in June, July and August.

Cyanobacterial species	Morphology	Mean size (μm)		
		June	July	August
*Aphanizomenon gracile*	Straight filaments	63 ± 40	116 ± 72	148 ± 55
*Aphanizomenon klebahnii*	Straight filaments	95 ± 35	103 ± 43	107 ± 46
*Dolichospermum crassum*	Spiral filaments	62 ± 25	77 ± 45	70 ± 24
*Dolichospermum flos-aquae*	Spiral filaments	53 ± 27	39 ± 16	36 ± 19
*Dolichospermum lemmermanii*	Spiral filaments	52 ± 21	41 ± 13	37 ± 13
*Microcystis botrys*	Colonies	113 ± 38	103 ± 51	98 ± 34
*Microcystis viridis*	Colonies	108 ± 57	101 ± 60	94 ± 41
*Microcystis wesenbergii*	Colonies	87 ± 41	92 ± 49	71 ± 46
*Planktothrix agardhii*	Straight filaments	212 ± 93	326 ± 162	297 ± 145

**Fig 2:**
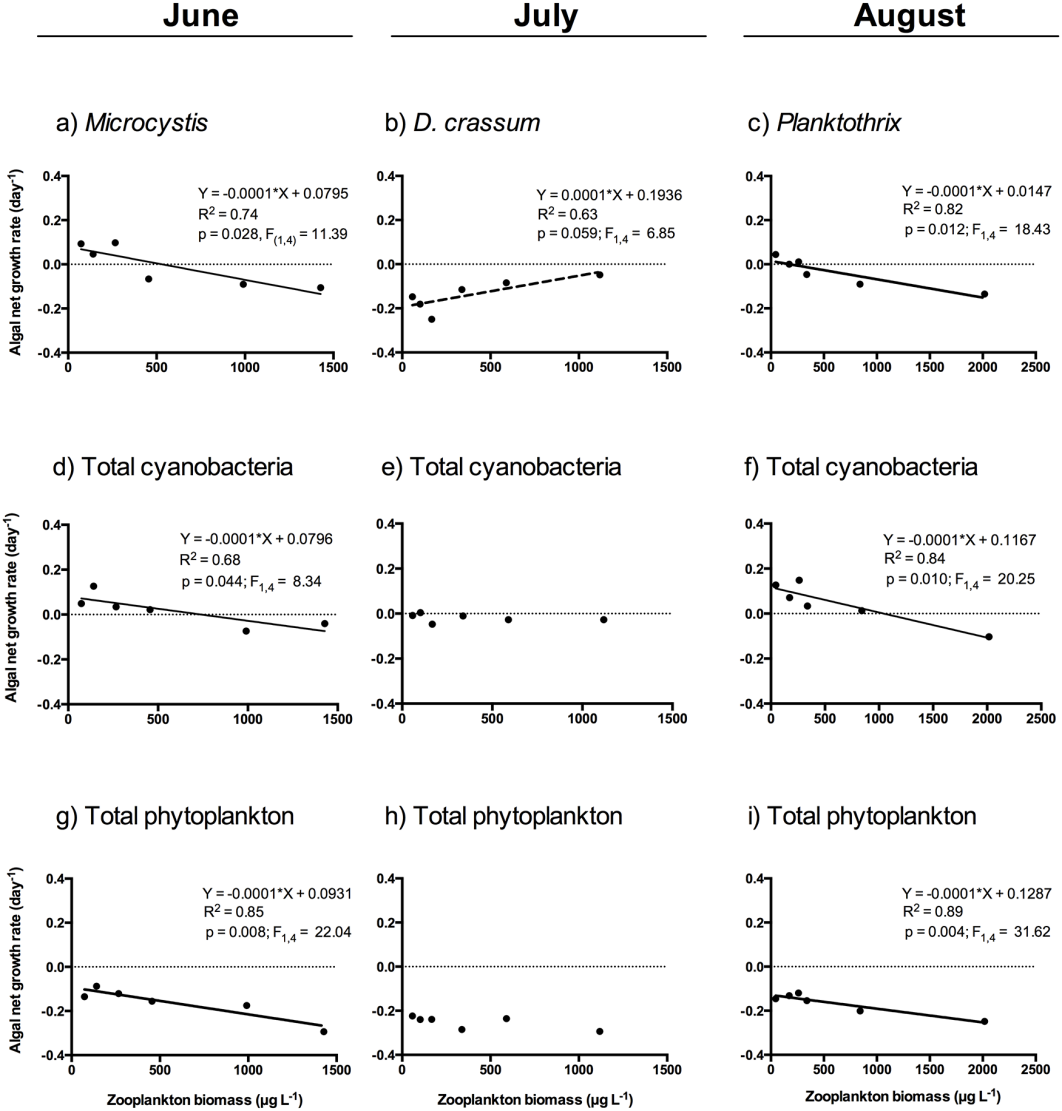
Example responses of selected cyanobacterial taxa (a-c), total cyanobacteria (d-f) and total phytoplankton (g-i) to the increasing gradient of *Daphnia* biomass during the grazing experiments conducted in June, July and August in the eutrophic lake Ringsjön. Fitted regression lines (n = 6), model equations, *F*-values, *R*^*2*^ values and *P*-values are given for significant linear relationships (*P* < 0.05, solid line). Note that for *Dolichospermum crassum* (July experiment) there was a marginally significant positive linear relationship (*P* = 0.059, broken line).

The large-bodied *Daphnia* significantly suppressed the net growth rate of the total phytoplankton community (measured as chlorophyll-*a*) in June and August, but not in July (Table 2, Fig 2). The same was observed for the total cyanobacterial biomass, even though cyanobacterial biomasses were substantially lower in June (Table 2, Fig 2). Although some cyanobacterial species (*Aphanizomenon gracile* and *Microcystis botrys*) showed a negative response to grazing in July (Table 2), these results coincided with a positive effect of grazer abundance on the net growth rate of the most abundant species *Dolichospermum crassum* (marginally significant, p = 0.059, Table 2, Fig 2). The net growth rate of *D*. *crassum* was never suppressed by *Daphnia* in any of the experiments (Table 2), whereas smaller filamentous species, such as *Dolichospermum flos-aquae* and *Dolichospermum lemmermanii*, were negatively affected by grazing in June and August (Table 2). The net growth rates of the genera *Aphanizomenon*, *Microcystis* and *Planktothrix* were negatively affected in one or more experiments (Table 2). The colony-forming *Microcystis botrys* was the species most vulnerable to grazing; the net growth rate of *M*. *botrys* showed a negative response to the presence of large-bodied *Daphnia* in the three experiments (Table 2).

**Table 2:** Responses of total phytoplankton (chlorophyll-a) and cyanobacteria at different levels of taxonomic resolution (species, genus, class), to *Daphnia* grazing in each of the experiments performed in June, July and August.

Algal community		June experiment					July experiment					August experiment				
		R^2^	F-value (_1,4_)	Equation	*P*-value	Response	R^2^	F-value (_1,4_)	Equation	*P*-value	Response	R^2^	F-value (_1,4_)	Equation	*P*-value	Response
**Species**	*Aph*. *gracile*	0.59	5.67	NS	0.076	−	0.87	27.65	Y = -0.0002*X + 0.0989	0.006**	−	0.78	13.93	Y = -0.0002*X + 0.2580	0.020*	−
	*Aph*. *klebahnii*	0.45	3.27	NS	0.145	−	0.19	0.95	NS	0.386	+	0.78	14.24	Y = -0.0001*X−0.1113	0.020*	−
	*D*. *crassum*	0.25	1.3	NS	0.318	−	0.63	6.85	Y = 0.0001*X−0.1936	**0.059†**	+	0.43	3.07	NS	0.155	−
	*D*. *flos-aquae*	0.68	8.58	Y = -0.0004*X + 0.2339	0.044*	−	0.34	2.04	NS	0.226	+	0.76	12.64	Y = -0.0002*X + 0.0532	0.024*	−
	*D*. *lemmermanii*	0.58	5.63	NS	0.078	−	0.32	1.88	NS	0.242	−	0.89	32.45	Y = -0.0003*X + 0.1005	0.006**	−
	*M*. *viridis*	0.35	2.2	NS	0.212	−	0.25	1.33	NS	0.314	−	0.28	1.56	NS	0.28	−
	*M*. *wesenbergii*	0.37	2.31	NS	0.203	−	0.35	2.17	NS	0.215	+	0.11	0.51	NS	0.514	−
	*M*. *botrys*	0.66	7.81	Y = -0.0000*X + 0.2673	0.049*	−	0.9	36.03	Y = -0.0010*X + 0.3072	0.004**	−	0.67	8.04	Y = -0.0003*X + 0.2884	0.048*	−
	*P*. *agardhii*	0.04	0.17	NS	0.703	+	0.117	0.531	NS	0.506	−	0.82	18.43	Y = −0.0001*X + 0.0147	0.012*	−
**Genus**	*Aphanizomenon*	0.81	17.44	Y = -0.0002*X + 0.0301	0.014*	−	0.31	1.83	NS	0.247	−	0.78	14.49	Y = -0.0001*X + 0.1709	0.020*	−
	*Dolichospermum*	0.35	2.17	NS	0.215	−	0.56	5.03	NS	0.088	+	0.28	1.59	NS	0.276	−
	*Microcystis*	0.74	11.39	Y = -0.0001*X + 0.0795	0.028*	−	0.49	3.85	NS	0.121	−	0.58	5.58	Y = −0.0004*X−0.0068	0.078	−
	*Planktothrix*	0.04	0.17	NS	0.703	+	0.117	0.531	NS	0.506	−	0.82	18.43	Y = −0.0001*X + 0.0147	0.012*	−
**Community**	Total cyanobacteria (a)	0.68	8.34	Y = -0.0001*X−0.0796	0.044*	−	0.1	0.454	NS	0.537	−	0.84	20.25	Y = -0.0001*X + 0.1167	0.010*	−
	Total phytoplankton (chlorophyll-*a*)	0.85	22.04	Y = -0.0001*X−0.0931	0.008**	−	0.49	3.84	NS	0.122	−	0.89	31.62	Y = -0.0001*X + 0.1287	0.004**	−

Probability levels of *F*-values of linear regression analyses (n = 6) are denoted in bold typing as follows:

* 0.05 < *P* ≥ 0.01

** *P* < 0.01

Equations are given for *P* < 0.05 (with exception of a marginally significant relationship for *Dolichospermum crassum* in the July experiment, *P* = 0.059†) and negative slopes indicate taxa that are suppressed by the grazers, and positive slopes indicate taxa that benefit from the grazer presence. For clarification we show the type of responses to grazing in a separated column based on the sign of the slopes from the linear regression analyses as: − (negative slopes) and + (positive slopes). Note that grazing effects were assessed on dominant cyanobacterial taxa present throughout the three experiments. Experimental data can be found in S1 File (Supporting Information). (a) Adapted from [35].

### Field study

**Fig 3:**
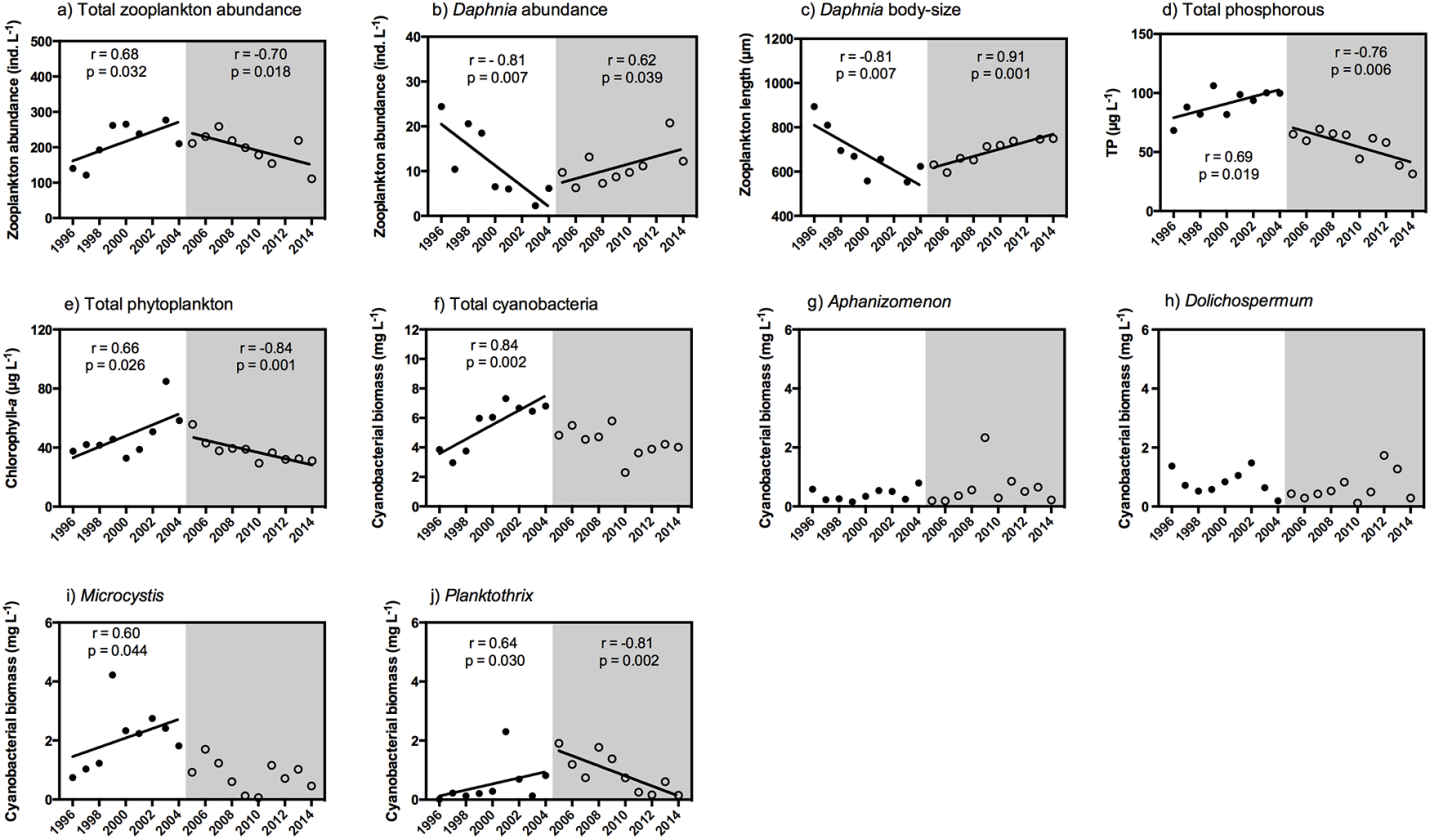
Temporal dynamics of chemical and biological variables prior to (1996–2004; filled circles and white background; n = 8, 9) and during biomanipulation in Lake Ringsjön (2005–2014; empty circles and grey background; n = 10). Values are seasonal means estimated from monthly values of the April-October period. Correlation coefficients are given as Pearson´s r and *P*-values for relationships (*P* < 0.05, solid lines) between each of the variables and time, both before and during the biomanipulation. Field monitoring data can be found in S1 File (Supporting Information).

The total zooplankton abundance in Lake Ringsjön (dominated by copepods and small cladocerans) increased from the mid-1990s until the start of the biomanipulation in 2005, after which total abundances of zooplankton leveled off and started to decline gradually (Fig 3). Fewer and smaller *Daphnia* individuals (dominated by *Daphnia cucullata* and *Daphnia galeata*) were found in Lake Ringsjön from 1996 to 2005, but the start of the biomanipulation in 2005 led to a change in the pattern with an increasing, positive trend in the abundance and body-size of *Daphnia* (Fig 3). Both total phosphorous and chlorophyll-*a* concentrations gradually declined during the biomanipulation (Fig 3). Similar to the total phosphorous and chlorophyll-a concentrations, the total cyanobacterial biomass significantly increased from 1996–2005, but a negative trend was observed after the start of the biomanipulation (Fig 3). While the filamentous cyanobacterial taxa *Aphanizomenon* and Dolichospermum did not show any apparent changes during the whole study period (Fig 3), the colony-forming *Microcystis* and filamentous *Planktothrix* showed an increasing trend in biomass until the start of the biomanipulation, after which the increasing trend shifted and they started to decline gradually (Fig 3).

## Discussion

Our experiments investigate the potential taxa-specific responses of cyanobacteria to zooplankton grazing in a desired biomanipulation scenario at low levels of fish predation, when large-bodied *Daphnia* tend to dominate zooplankton communities [7,17]. Our results show that large-bodied *Daphnia* can suppress the growth of several *Aphanizomenon*, *Dolichospermum*, *Planktothrix* and *Microcystis* bloom-forming species. Total phytoplankton and cyanobacterial biomasses were also significantly suppressed in early and late summer. Yet we found a positive response to zooplankton presence in the net growth rate of the filament-forming *Dolichospermum crassum* in the July experiment, when this species dominated the phytoplankton community. Some cyanobacterial filaments may interfere with and clog the filtering apparatus of *Daphnia*, which causes severe disturbance of the filtering process and affects the ingestion of other algae [10]. Although we cannot rule out the involvement of chemical defenses or other unknown mechanisms, the specific morphology of *Dolichospermum crassum* might have affected the grazing efficiency of *Daphnia magna* through mechanical disturbance of filtering, which would explain why total phytoplankton and cyanobacterial biomasses were not reduced in July. This suggestion is consistent with the fact that, despite increased grazing rates by cladocerans with larger body-size [35], herbivory by *Daphnia* can still be inhibited by certain cyanobacterial species [10,12]. The fact that feeding inhibition by cyanobacterial filaments also increases with *Daphnia* body-size [11], poses important questions on the use of food web manipulations that facilitate the dominance of large-bodied *Daphnia* [7]. This demands further studies investigating the mechanisms behind the different responses of cyanobacterial species to grazing by large-bodied *Daphnia*, which is crucial to determine the effectiveness of these restoration practices.

Resource densities, which differed considerable between the June and August experiment, apparently did not affect the herbivore effects of large-bodied *Daphnia* on the net growth rate of cyanobacterial species. Small-bodied zooplankters that generally coexist with cyanobacterial blooms, such as copepods and small cladocerans, are more selective herbivores and may feed on the nutrient-poor cyanobacteria, but only when little alternative food is available [15]. However, *Daphnia* are generalist filter feeders that indiscriminately ingest particles as they encounter them while swimming [8]. The capacity of *Daphnia* to suppress cyanobacterial growth shown in this study, when their relative contribution to total phytoplankton biomass is low, may have important implications for cyanobacterial bloom management; for example, intense grazing upon cyanobacteria when they recruit from sediments early in the season and remain at low densities may restrict later bloom formation and development [14,36].

Two of three species of *Dolichospermum* differed in their vulnerability to grazing by *Daphnia*. Although chemical defenses could also be involved in this response [8], these results suggest that the small filament size of *D*. *flos-aquae* and *D*. *lemmermanii* increased their grazing vulnerability with respect to *D*. *crassum*. On the other hand, *Aphanizomenon* and *Planktothrix* species showed considerable susceptibility to grazing by *Daphnia* in spite of presenting the largest sizes of filaments. The size of such cyanobacterial species surpasses the optimum size range for food given the body-size of the *Daphnia* used in the experiments [35]. However, cyanobacterial species also showed a large variation in size (Table 1), which likely made it possible for *Daphnia* to access a substantial part of their populations consisting of small filaments. In addition, unlike the *Dolichospermum* species present in this study, the trichomes of *Aph*. *gracile*, *Aph*. *klebahnii* and *P*. *agardhii* did not display spirals and curvatures that could also be involved in defense against grazing. Interestingly, the growth of *Microcystis botrys* was suppressed by *Daphnia* in all experiments, even though this species accounted for a 300% increase in cyanotoxins (microcystins) in a previous experiment [31]. Also, in a survey of lakes throughout Europe [37], this species was identified as having the highest proportion of toxic genotypes (90%) compared to other cyanobacteria—that is, colonies containing microcystins and the gene *mcy* responsible for its synthesis. Microcystins are potent hepatotoxins that may affect the growth and fitness of herbivores negatively [14,15]. These findings contrast with the results obtained by [24], who showed that cyclopoid copepods and small cladocerans always boosted the growth of *M*. *botrys*. We identified relevant microcystin concentrations in these experiments (see [23]) and the fact that the growth of potential toxic species, such as *M*. *botrys*, was always suppressed by *Daphnia* highlights the importance of large, generalist feeders as a way to control toxic cyanobacterial blooms. In addition, in eutrophic systems *Daphnia* can evolve tolerance to toxic cyanobacteria [38], which may enhance grazer control of cyanobacteria, thereby counteracting (though with a metabolic cost; [8]) the potential toxic effects of cyanobacteria on *Daphnia* fitness, reproduction and survival [39–40].

Our field study demonstrates that biomanipulation by reducing fish predation can be used to increase the abundance (50% increase) and body-size (20% increase) of *Daphnia* herbivores (Fig 3). Chlorophyll-a levels and total cyanobacterial biomass showed a decreasing trend since the start of the biomanipulation (Fig 3), as did the two most dominant cyanobacterial taxa, *Microcystis* and *Planktothrix* (Fig 3). Hence, this increased dominance of *Daphnia* likely resulted in stronger top-down control on the phytoplankton and cyanobacterial community [17]. Studies in Lake Ringsjön have shown that such cascading effects of biomanipulation on *Daphnia* dominance and cyanobacterial biomass are stronger preceding the hatching of 0+ fish in summer [23,41]. Alternatively, biomanipulation may also increase grazing pressure by other zooplankters (e.g., cyclopoid copepods) on specific cyanobacterial taxa during summer, observations that our field study here cannot capture (but see [24]). In addition, total phosphorous levels decreased with the biomanipulation (Fig 3), likely due to reduced re-suspension of nutrients from the sediments by removing benthic-feeding fish (such as bream) [41] and this likely contributed to limiting cyanobacterial growth. Hence, trophic cascades may affect phytoplankton community structure and ecosystem functioning through various complex mechanisms, aside from zooplankton grazing alone, including nutrient availability or alterations in nutrient recycling patterns [42]. Although our field data do not allow quantification of the relative effects of nutrients and zooplankton grazing, our experiments show clearly that larger-bodied *Daphnia* (> 1500 μm mean body-size), expected to dominate at lower levels of fish predation, can inflict a strong species-specific control on many cyanobacteria, including 66% of the tested *Aphanizomenon*, *Dolichospermum*, *Microcystis* and *Planktothrix* species. This is important because residual populations of large-bodied *Daphnia*, such as *Daphnia magna*, can persist in many eutrophic lakes [43–45]. This suggests that there is the potential to boost their dominance in these systems, although this will depend on our capacity to foster further research to improve current methods for controlling fish predation [7].

Our results have important implications not only for understanding consumer-prey interactions, but also for lake restoration practices to improve the water quality of eutrophic systems. Our grazing experiments exemplify the potential, complex responses that may emerge among different cyanobacterial taxa in response to zooplankton grazing; the net growth rate of algae can either increase or decrease to elevated zooplankton grazing depending on compensatory effects of ingestion and nutrient recycling [26,27]. In this sense, would a zooplankton community dominated by larger-bodied *Daphnia* be capable of controlling cyanobacterial growth, given a scenario of more limited fish predation than the currently achieved in Lake Ringsjön? Despite effective grazing by large-bodied *Daphnia* on many cyanobacterial species, as well as on the total cyanobacterial and phytoplankton community, the marginally significant increase in the net growth rate of *Dolichospermum crassum* in one of the three experiments suggests that some species possess reduced vulnerability to grazing by large-bodied *Daphnia*. In contrast to the experiments, the putative grazing-resistant cyanobacterium *Dolichospermum* (dominated by *D*. *crassum*) did not increase in response to the decrease in biomass of the other cyanobacterial competitors (*Microcystis* and *Planktothr*ix) following the biomanipulation in the Lake Ringsjön. This contrast with our experimental results and the fact that *Daphnia* in the lake were dominated by smaller-bodied species (*D*. *cucullata* and *Daphnia galeata*) than *Daphnia* magna, which possess lower grazing rates on phytoplankton and are possibly less capable to control cyanobacterial growth [8]. Hence, in addition to the stronger top-down pressure on cyanobacteria by about 50% more abundant and 20% larger *Daphnia* compared to before the biomanipulation, this suggests that a reduction in the availability of nutrients likely contributed to the growth limitation of cyanobacteria, especially in grazing-resistant species such as *D*. *crassum*. Altogether, these results indicate that managing cyanobacterial blooms solely based on the grazer control by large-bodied *Daphnia* is, nonetheless, challenging, as it should overcome cyanobacterial defenses, such as clogging of the filtering process [12], other potential defensive toxic and nutritional constraints [13–15] and a very strong reduction in fish predation pressure [7]. Although there are numerous cases of biomanipulation success [7,22], our results are consistent with the idea that grazer control of harmful cyanobacteria could be secured more effectively when other restoration methods proven to be effective (e.g., nutrient reductions) are conducted in conjunction [7].

In conclusion, here we show both the capacity of large-bodied *Daphnia* to graze on a wide range of bloom-forming cyanobacterial species and the potential use of biomanipulations (via fish removal) to enhance the abundance and body-size of such *Daphnia* herbivores. However, we also identified a grazing-resistant species in our experiments, which indicates that, in a desired biomanipulation scenario at lower levels of fish predation in the lake, the food web facilitation of large-bodied *Daphnia* may not be sufficient to control the overgrowth of all the cyanobacterial diversity. In addition, we have to acknowledge that our experimental findings are based on the methods from [26] and others [27–29], which are short-lasting experiments. These experiments are effective because they provide an effective screen of zooplankton grazing rates on phytoplankton, by allowing changes in phytoplankton community composition, while zooplankton biomass can be held constant and enclosure effects are minimal. However, we also encourage performing follow-up studies over longer periods to determine whether large-bodied *Daphnia* are capable of maintaining similar grazing rates on cyanobacterial species that were identified susceptible to grazing. For example, top-down control of cyanobacteria by *Daphnia* may sometimes weaken over several generations [46], which again supports the conclusion that management practices should include other means of controlling cyanobacterial growth. Overall, our results provide important knowledge on taxa-specific responses of cyanobacteria to the grazer control by large-bodied *Daphnia*. More evaluations as these are needed to facilitate predicting restoration practices aimed to improve the ecological and societal status of eutrophic lakes through the food web facilitation of large-bodied *Daphnia* grazers.

## Supporting Information

S1 File Excel file with experimental and field data for this manuscript. Experimental data includes zooplankton biomasses, chlorophyll-*a* and cyanobacterial biomasses at different level of taxonomic resolution (community, genera and species level), as well as calculated algal net growth rates along the gradient of zooplankton biomass for each experiment conducted in 2012 in Lake Ringsjön. Field data includes monthly monitoring values (April-October from 1996 to 2014) for total zooplankton abundances, *Daphnia* abundances and body-size, chlorophyll-*a*, total phosphorous, and biomasses of the total cyanobacterial community and dominant cyanobacterial genera in Lake Ringsjön.
(XLSX)
